# A New Model of Air–Oxygen Blender for Mechanical Ventilators Using Dynamic Pressure Sensors

**DOI:** 10.3390/s24051481

**Published:** 2024-02-24

**Authors:** Gabryel F. Soares, Gilberto Fernandes, Otacílio M. Almeida, Gildario D. Lima, Joel J. P. C. Rodrigues

**Affiliations:** 1 Department of Electrical Engineering, Federal University of Piauí (UFPI), Teresina 64049-550, Brazil; gabryelfsoares@ufpi.edu.br (G.F.S.); otacilio@ufpi.edu.br (O.M.A.); 2 Computer Science Department, State University of Londrina (UEL), Londrina 86057-970, Brazil; gilfernandes6@gmail.com; 3 Federal University of Delta do Parnaíba (UFDPar), Parnaíba 64202-020, Brazil; gildario@ufpi.edu.br; 4 COPELABS, Lusófona University, 1749-024 Lisbon, Portugal

**Keywords:** blender, *FIO*_2_, mechanical ventilator, simulation

## Abstract

Respiratory diseases are among the leading causes of death globally, with the COVID-19 pandemic serving as a prominent example. Issues such as infections affect a large population and, depending on the mode of transmission, can rapidly spread worldwide, impacting thousands of individuals. These diseases manifest in mild and severe forms, with severely affected patients requiring ventilatory support. The air–oxygen blender is a critical component of mechanical ventilators, responsible for mixing air and oxygen in precise proportions to ensure a constant supply. The most commonly used version of this equipment is the analog model, which faces several challenges. These include a lack of precision in adjustments and the inspiratory fraction of oxygen, as well as gas wastage from cylinders as pressure decreases. The research proposes a blender model utilizing only dynamic pressure sensors to calculate oxygen saturation, based on Bernoulli’s equation. The model underwent validation through simulation, revealing a linear relationship between pressures and oxygen saturation up to a mixture outlet pressure of 500 cmH_2_O. Beyond this value, the relationship begins to exhibit non-linearities. However, these non-linearities can be mitigated through a calibration algorithm that adjusts the mathematical model. This research represents a relevant advancement in the field, addressing the scarcity of work focused on this essential equipment crucial for saving lives.

## 1. Introduction

The COVID-19 pandemic exposed the fragility of healthcare systems worldwide, particularly regarding resource scarcity and the management of critically ill patients. The overwhelming demand for mechanical ventilation, a life-saving technology for severe respiratory cases, highlighted critical limitations in equipment availability and performance. One crucial element in these ventilators, the air–O2 blender, proved susceptible to challenges like imprecise adjustments, flow sensor limitations, and efficiency decline with cylinder pressure depletion. These issues directly impacted patient care and outcomes, particularly during the peak of the pandemic when resources were most stretched.

Instances like the one witnessed in the state of Amazonas, Brazil served as an illustration of the severity of the situation in the absence of adequate resources or planning. The critical shortage of oxygen cylinders throughout the state marked the peak of the ensuing chaos, resulting in multiple fatalities among hospitalized patients [1,2,3].

One crucial tool in the battle against COVID-19 is the utilization of mechanical ventilation to address severe cases. This vital equipment, found in ICUs for patients with comorbidities, administers a controlled air–oxygen mixture with regulated pressure and volume. Its function varies, acting as a complement to the patient’s breathing or fully taking over, depending on the severity of the situation. The structure of a mechanical ventilator is relatively simple, comprising an air–oxygen blender, unidirectional valves, a microcontroller system for parameter control and adjustments, a breathing circuit, and an integrated humidifier [4,5,6]. Despite its critical role in saving lives, prolonged use or imprecise adjustments in mechanical ventilation are associated with various physical and mental issues in patients post-ICU discharge. Therefore, the manufacturing process must follow rigorous standards with no room for error [7,8].

The air–O_2_ blender stands out as the central component in any mechanical ventilation equipment. Tasked with regulating the air and O_2_ proportions for patients, thereby controlling the inspiratory oxygen fraction (FiO_2_), it can be constructed in various ways, such as a Venturi tube, Poppet-configuration area asset variation, three-terminal solenoid valves, or electronic devices. Typically, a blender includes an air inlet, an O_2_ inlet, a bypass system for excessive pressures, a gas equilibrium stage, and a mixing stage [9,10,11,12]. Previous studies have evaluated similar aspects on Venturi, turbine, or mixer systems for non-invasive ventilation [13,14].

At the air and oxygen inlet, filters are in place to prevent contamination, and one-way valves ensure that the gas flow does not return. The bypass system relies on the pressure difference between the two gases entering the equipment. If this limit is surpassed, an audible or visual alarm is triggered, and all gas is redirected to a valve, releasing it into the surrounding air. Balancing is a critical step since gases can pass through the valves at varying pressures; thus, balancing is set to the lowest inlet pressure. The mixing stage involves a chamber where the two gases, now equalized, enter. This mechanism is operator-adjusted, allowing the operator to define the percentage of oxygen, and whereas blenders commonly feature manual adjustment, applications with automatic controllers are also available. Feedback on the percentage of the air–oxygen mixture is not always present, and when available, it typically comes through flow sensors [15,16,17,18].

The precise regulation of oxygen concentration has a direct impact on patient outcomes. Delivering too little oxygen risks organ failure, whereas excessive oxygen can be toxic. Current blenders, however, have limitations. They often rely on expensive flow sensors, increasing healthcare costs. Manual adjustments introduce a potential for error, impacting the accuracy of oxygen delivery. Additionally, these blenders often perform poorly at low pressure, potentially compromising care for critically ill patients on reduced ventilator settings. Addressing these limitations through innovative blender designs, such as sensors with improved cost-effectiveness and automated adjustments, could significantly improve patient care and outcomes, especially in resource-constrained settings [15,16,17,18].

Thus, this paper proposes a model for a new micro-controlled air–O_2_ blender with output oxygen saturation determination based on the measurements from three pressure sensors. The main contributions of this paper include:

- Development of a mathematical model to calculate oxygen saturation using only measurements from three pressure sensors, considering their cost-effectiveness and significantly shorter response time compared to flow sensors;
- Proposal for optimizing the utilization of air and oxygen cylinders through the mathematical model and adjustment of valves during use;
- Introduction of a blender methodology designed to operate effectively at low pressures.

This paper is organized as follows. Section 2 provides a summary of the main contributions related to the subject under study, aiming to highlight the innovation proposed in the article. Section 3 demonstrates the proposed mathematical model and presents how the simulation study was performed and its properties, as well as the algorithm used. Section 4 presents the outcomes obtained by applying the proposed method in the simulation study. Section 5 focuses on the analysis and discussion of obtained results and, finally, Section 6 concludes the paper by summarizing the key findings and implications of this research while suggesting the next steps towards this study.

## 2. Related Work

Due to its limited exploration, there is not much research on technological advancements for air–O_2_ blenders. With scarce and sometimes outdated references, this limitation has posed challenges in researching the theoretical foundation, making this work important in addressing this crucial topic.

The study conducted by Ng et al. [19] evaluates a specialized low-flow blender (0.5 to 4.0 L/min) designed for children up to 5 years old, offering safety, efficiency, and affordability. Operating on the Venturi principle, the blender regulates a mixture of pressurized oxygen gas and air through adjustments within the equipment body. Notably, its unique feature allows for the use of nasal cannulas, reducing gas resistance during delivery. Test results demonstrated oxygen concentration within 0.5% of the desired value and accuracy within 0.1 L/min of the target flow. However, due to its low-flow limitation and manual adjustment, it may not be suitable for severe illnesses and may lose accuracy over time due to wear and tear. Introducing electronic valves and a methodology based on pressure sensors, as proposed in this paper, enhances safety and durability by precisely adjusting moving parts.

Similar to the previous study, the study by Mollazadeh et al. [20] introduces a Venturi tube-based blender; although cost-effective and capable of adjusting $FiO_{2}$, this method relies heavily on precise manufacturing and adjustments due to its millimeter-scale openings. In contrast, electronically controlled blenders offer greater user and medical team flexibility, reducing precision concerns. Nevertheless, the crucial principle of affordability guides efforts to enhance equipment accessibility, particularly in under-served regions, through techniques such as 3D printing and resource optimization.

The equipment introduced by Mukkundi et al. [21] emphasizes simplicity, affordability, and efficiency. With pressure capabilities of 0–8 cmH_2_O and a capacity of up to 5.5 L per minute, it offers variable $FiO_{2}$ ranging from 21% to 100% based on oxygen flow. Utilizing an oxygen cylinder and atmospheric air pump as gas sources, it achieves satisfactory results. However, limitations include inability to handle high pressures and manual $FiO_{2}$ adjustments prone to inaccuracies over time. Implementing the methodology proposed in this article could enhance precision and provide more electronic data for improved performance.

A key challenge with blenders lies in gas measurement accuracy, particularly for mixed gas. According to Dion et al. [22], comparing flow measurements from blender regulators with those from mechanical ventilators reveals a margin of error, particularly pronounced in simpler, analog blenders. In some configurations, the actual flow can exceed the indicated value by up to 1 L, posing significant risks, especially for newborns. Therefore, the implementation of electronics and the proposed methodology based on pressure sensors is crucial, offering greater accuracy and instantaneous readings compared to gas sensors.

The blender proposed by [23] consists of a microcontroller system that automatically adjusts the electronic valves that regulate the mixture of gases. All of this is based on readings from flow and gas sensors, supplying the patient according to needs. Despite all this, the use of flow and gas sensors can be a limiting factor in the development of the equipment. Thus, the implementation of the model presented in this article using pressure sensors would significantly improve the process of development and evolution of this system.

Privitera et al. [13] aimed to evaluate the impact of filters on the delivered fresh gas flow, $FiO_{2}$ levels, and noise levels during helmet-CPAP administered by three different flow generators. Authors conducted bench experiments using an air–oxygen blender, turbine ventilator, and Venturi system to deliver CPAP at varying gas flow rates and fixed $FiO_{2}$ levels, with different PEEP settings. Results showed significant differences in flow variation among the generators after filter application, with the Venturi system exhibiting the highest flow reduction and a significant $FiO_{2}$ variation post-filter application.

Another interesting bench study was conducted by Capsoni et al. [14]. They assess the performance of four Venturi devices in delivering helmet-CPAP with clinically relevant gas flow, $FiO_{2}$, and PEEP values using a single oxygen cylinder. Three double-inlet Venturi systems and one direct attachment system were evaluated, with results showing that EasyVent, Ventuplus, and O2-MAX successfully delivered helmet-CPAP setups, whereas Compact-HAR did not. The study concludes that portable Venturi systems can be used to provide helmet-CPAP, but not all are suitable.

The use of automated controllers also showed great promise when applied to the air–O_2_ blenders. Techniques such as PID and Fuzzy are considered the best options for this type of adjustment. Speaking of percentages, closed-loop controllers improve by approximately 63% over manually tuned devices. This reflects a better quality of life for the patient, less chance of error by the health team, and greater optimization of the resources used in the treatment. In order for the control algorithms to operate effectively, it is necessary to have accurate information about the oxygen saturation levels in the mixed gas. Typically, it is very common to use gas flow sensors, but this brings high costs and difficulty in finding such devices, mainly due to the COVID-19 pandemic [24,25,26]. However, the proposed methodology in this study is based on pressure sensors, which offers a cost-effective alternative. By utilizing pressure sensors instead of gas sensors, implementing control algorithms becomes more feasible, leading to cost reduction and overcoming the challenges posed by sensor availability.

To further demonstrate this paper’s approach, the materials and methods used in developing the mathematical model for calculating oxygen saturation are presented below in detail.

## 3. Materials and Methods

### 3.1. Mathematical Model Proposal

Based on the presented problem and the state of the art, this section presents the developed equation for calculating oxygen saturation using the Bernoulli equations.

Firstly, the study of gases in motion is governed by the Bernoulli equation under ideal conditions, as stated below:

(1) $$P=P_{0}+\rho gh+\frac{1}{2}\rho v_{0}^{2}=const$$

**Figure 1:**
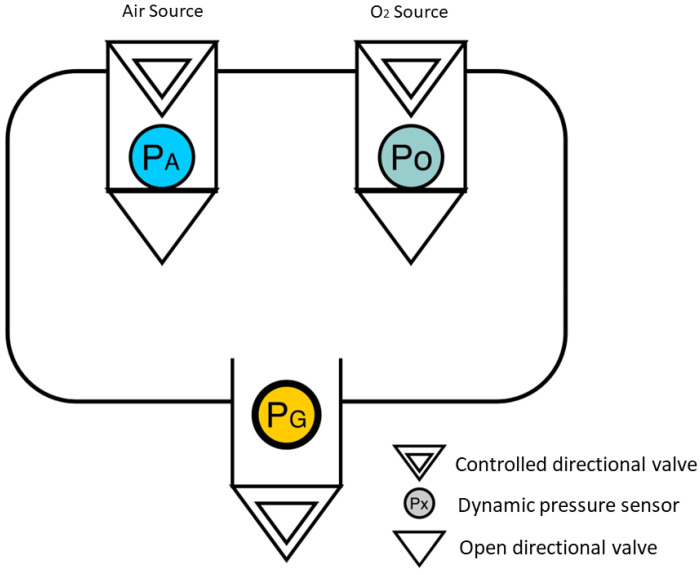
Flowchart of a blender from three pressure sensors. (Air Source in blue ($P_{A}$), Oxygen source in green ($P_{O}$) and a general pressure sensor in yellow ($P_{G}$)).

Considering the problem described by Figure 1, there is a representation that supports the methodology of a blender built from three pressure sensors. The diagram represents a container with two controlled gas inputs, oxygen and air (Points A and B), and a controlled mixing output (Point C).

The main interest lies in deriving a saturation function ($S_{O}$) from real-time measurements of the inlet pressures of air and oxygen:

(2) $$S_{O}=S\left(P_{A},P_{O}\right)$$

To begin the derivation of Equation (2), which will regulate the model, the following conditions must be considered:

- A—Both gases will be submitted to a regime of low pressures compared to the ambient pressure;
- B—The difference in pressure resulting from the difference in heights between points A, B, and C will be negligible;
- C—The sum of the amount of volume that enters the bulge is the same that leaves the bulge in time; that is:
  (3) $$Q_{G}=Q_{A}+Q_{O}$$
  where $Q_{n}$ is the flow, defined by:
  (4) $$Q_{n}=\rho_{n}v_{n}A$$
  and $\rho_{n}$ is the gas density, $v_{n}$ the gas velocity, and *A* is the cross-sectional area of the free valves (one-way release valves).
- D—Within the output pressure regime (General Pressure-$P_{G}$ or Working Pressure), it is possible to assume that due to item B, the gas flow velocity ($v_{n}$) is the same for all gases.

To delve into the development of the mathematical model, it is necessary to justify certain approximations. The initial consideration is that the gases employed in the system are oxygen and ambient air. Key points to address include the definition of the function $S_{O}$, which is determined by the oxygen flow within the mixture in relation to the total gas flow exiting the blender:

(5) $$S_{O}=\frac{\left(Q_{O}+\kappa Q_{A}\right)}{Q_{G}}$$

Here, $Q_{O}$ represents the oxygen flow, $Q_{A}$ represents the flow of ambient air, and $Q_{G}$ represents the total gas flow. By considering that even ambient air contains a fraction κ of oxygen contributing to the total saturation, this equation appropriately captures the oxygen content within the mixture.

Furthermore, it is observed that even air contains a κ fraction of oxygen that also contributes to the total saturation. So, by applying the proposed conditions to Equation (1), we derive the following equation, which further refines the definition of oxygen saturation $S_{O}$.

(6) $$S_{O}=\frac{\left(P_{O}+\kappa P_{A}\right)}{\left(P_{O}+P_{A}\right)}$$

In Equation (6), $P_{O}$ and $P_{A}$ represent the partial pressures of oxygen and ambient air, respectively, and κ represents the fraction of oxygen in ambient air. This equation provides a more precise calculation of oxygen saturation based on the partial pressures of oxygen and ambient air in the mixture.

One distinctive feature of the proposed methodology is the inclusion of a third pressure sensor at the outlet, which can offer a controlled working pressure as an additional safety measure to prevent damage, such as barotrauma. This way, once the working pressure ($P_{G}$) of the blender has been established, it can be correlated with the following equation:

(7) $$P_{G}=P_{A}+P_{O}$$

and, therefore, the air inlet pressure is defined from $P_{O}$, allowing us to rewrite Equation (6) in the form:

(8) $$S_{O}=\frac{P_{O}\left(1-\kappa \right)}{P_{G}}+\kappa$$

An important aspect to consider is the decision to employ a baseline output pressure of 70 cmH2O in constructing the model. This choice was made to ensure the model’s capability to operate with pressures surpassing the blender’s safety limit, typically set at 35 cmH2O. Adopting this approach guarantees a sufficient supply of pressure beyond the safety threshold, thereby enabling the model to accommodate a wider range of scenarios and operational conditions.

### 3.2. Simulation Study

Furthermore, to validate the model and explore the dynamics of the blender, a simulation study was conducted using the SimCenter AMESim software v2304, incorporating the “gas mixture” library.

**Figure 2:**
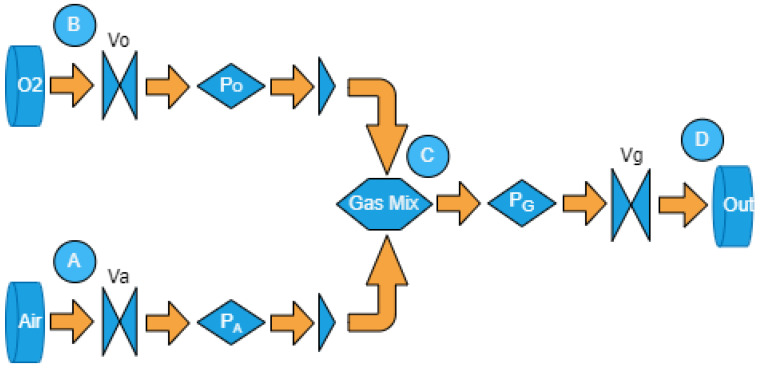
Model simulation diagram.

Three dynamic pressure sensors and three electro-controlled switches were used. The blender model is based on Figure 1, and its didactic diagram is illustrated in Figure 2, featuring the following properties: (I) Electrically controlled unidirectional valves, namely $V_{a}$, $V_{o}$, and $V_{g}$, which can be positioned anywhere between 100% open and 100% closed; (II) Valves located at points *A* and *B*, receiving air and $O_{2}$ from constant-pressure repositories, with no pressure drops in relation to the system load; (III) Dynamic pressure sensors after valves $V_{a}$ and $V_{o}$, capable of estimating pressure differences at each time step *n* during readings. Each simulation step corresponds to a 30 s time interval. Post-sensors, unidirectional valves are employed to prevent the backflow of gaseous flow; (IV) The equation governing saturation is referenced by Equation (8); (V) Input parameters include $P_{G}$ (output pressure) and the dimensions of components in the simulator; (VI) Reading parameters consist of $P_{A}$ and $P_{O}$; (VII) Control parameters involve the microcontroller-operated valves $V_{A}$, $V_{O}$, and $V_{G}$; (VIII) The mixing chamber is denoted by point *C*, and the mixture’s output to the fan is represented by point *D*.

The algorithm developed to conduct the simulations initially involves determining the maximum opening of $V_{o}$ to ensure that the value of $P_{g}$ remains within the defined reference, with $V_{a}$ set at 100% closed. The same procedure is then repeated for $V_{a}$, but this time with $V_{o}$ set at 100% closed. This iterative process is replicated for *n* arbitrary values of $P_{g}$.

In the subsequent steps, for each $P_{g}$, $V_{o}$ is set to its maximum opening value determined at the beginning of the algorithm, whereas $V_{a}$ is kept completely closed. Following this, a series of steps are executed to gradually close $V_{o}$ while simultaneously opening $V_{a}$, where the maximum limit of $V_{a}$ is defined by the value found at the algorithm’s outset. It is noteworthy that $V_{g}$ is maintained at 50% opening throughout this process. This methodology allowed the capture of the dynamics of the system for various $P_{G}$ values.

In conclusion, additional rounds of simulation were conducted, but this time incorporated Equation (8) to determine oxygen saturation and a gas concentration sensor to validate the proposed mathematical model. The outcomes of this simulation study are detailed in the following section.

## 4. Results

**Figure 3:**
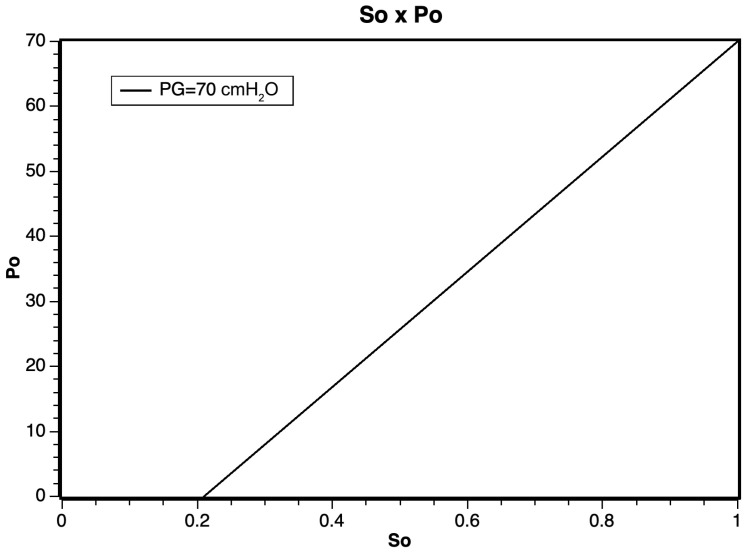
Application of the (8) as a function of the oxygen inlet pressure.

From the mathematical model in Equation (8), it was possible to obtain the estimated response graph, demonstrating a linear relationship within the working pressure regimes, as illustrated in Figure 3. However, for pressures exceeding 500 cmH_2_O, it is anticipated that the linear relationship may be impacted due to the effects of gas confinement and compression.

**Figure 4:**
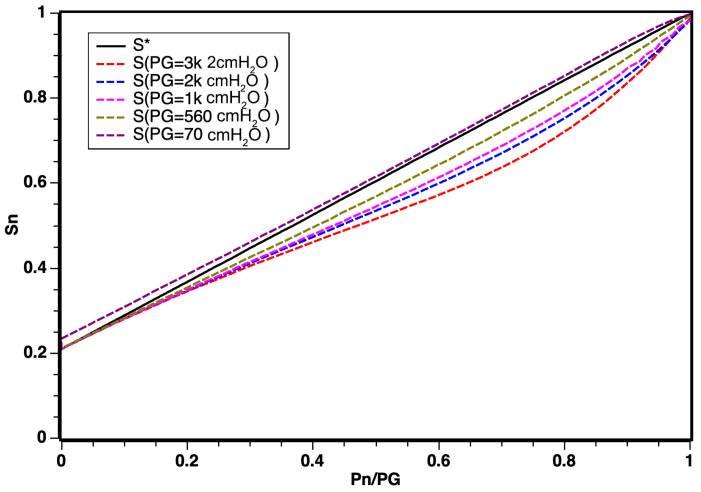
Distribution of $O_{2}$ saturation curves in relation to changes in $O_{2}$ pressure. Note that $S^{*}$ is the theoretical result from Equation (8).

After the mathematical modeling and the execution of the simulations, some results could be obtained. For the low-pressure regime, as shown in Figure 4, a linear regime between the oxygen pressure ($P_{O}$) and the oxygen saturation ($S_{O}$) of the mixture leaving the blender was expected.

After completing the mathematical modeling and conducting simulations, certain results were obtained. In the low-pressure regime, as depicted in Figure 4, a linear relationship between oxygen pressure ($P_{O}$) and oxygen saturation ($S_{O}$) in the mixture exiting the blender was expected.

**Figure 5:**
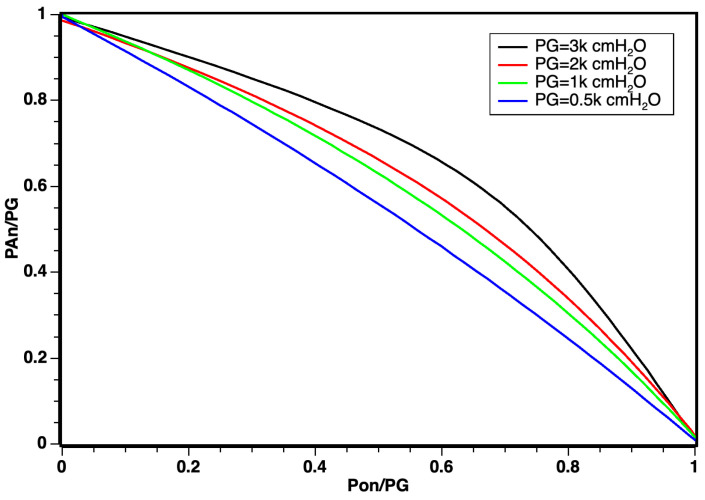
Distribution of dynamic inlet pressures for oxygen and air obeying the relation $P_{G}=P_{A}+P_{O}$.

According to simulation results illustrated in Figure 5, the linear regime demonstrated a robust response for a working pressure of $P_{G}$ = 500 cmH_2_O and that for values from $P_{G}$ up to 3.000 cmH_2_O, there was a slightly curved behavior, which can be explained by the effects of confinement and compression of gases at high pressures. The graph shows that, as the working pressure increased, the relationship between pressures ranging from $\left[0,P_{G}\right]$ for oxygen *P*_*O*_*n*__ and air *P*_*A*_*n*__, *n* being the various working pressures $P_{G}$, constitutes a new working regime.

However, for values ranging from $P_{G}$ up to 3.000 cmH_2_O, a slightly curved behavior was observed. This deviation can be attributed to the effects of confinement and compression of gases under high pressures. The graph indicates that, with increasing working pressure, a new operating regime emerges, portraying the relationship between pressures in the range $\left[0,P_{G}\right]$ for oxygen *P*_*O*_*n*__ and air *P*_*A*_*n*__, where *n* represents various working pressures $P_{G}$.

The curvature observed in Figure 5 does not negate the linear behavior results, especially considering that the optimal working pressure in modern respirators corresponds to $P_{G}$ = 500 cmH_2_O. The identification of this corrective curvature stands as a novel discovery for non-electrically controlled classic regime mixers equipped with valves and sensors.

## 5. Discussion

Advancing into the discussion regarding the results obtained in the preceding section, it is important to note that the curves underwent an audit by a simulator, aiming to approximate real-world scenarios. The intention is to conduct further experiments in future works to validate these findings in a laboratory setting and efficiently address the anticipated curvature due to the effects of gases under high pressure. This curvature effect can be recalibrated by up to 100% through the algorithm governing the reading dynamics. Once technical specifications are defined, the device will be calibrated to obtain a correction curve, ensuring the construction of a blender with controlled valves and pressure sensors that offer high precision for oxygen saturation and barometric protection for patients while controlling the working pressure $P_{G}$.

From the conducted study, we can substantiate the mathematical foundation derived from the proposed Bernoulli equation. It demonstrates that the curves correlating pressures undergo only slight deformation when the saturation $O_{2}$ is exposed to pressures much higher than $P_{G}$ = 70 cmH_2_O. The non-linearity observed for working pressures exceeding 500 cmH_2_O can be attributed to the potential effects of confinement and interactions resulting from the simulation’s dynamic pressures of oxygen and air. This effect can be entirely absorbed through mathematical modeling during the factory calibration of the blender.

This work has yielded significant results for the development of an air–oxygen blender: the determination of oxygen saturation solely from dynamic pressure sensors; whereas previous studies such as [19,21,23] have made valuable contributions to the field, they are not without limitations. For instance, ref. [19] is constrained by low-flow limitations and manual adjustment, whereas [21] faces challenges in handling high pressures and manual $FiO_{2}$ adjustments. Similarly, ref. [23] relies solely on readings from flow and gas sensors to supply the patient according to their needs. In contrast, the model proposed in this paper introduces electronic valves and a methodology based on pressure sensors, enhancing safety and durability by precisely adjusting moving parts. By implementing this methodology, precision can be improved, and more electronic data can be obtained for enhanced performance.

Furthermore, several advantages emerged throughout the research, including: (i) faster and more effective pressure measurements due to the intensive and time-efficient nature of pressure as a quantity; (ii) the implementation of the $V_{G}$ valve, a crucial safety measure in the patient’s intubation process, serving as an additional safeguard within the procedure; (iii) the utilization of electronically controlled valves offering enhanced automation and precision in adjustments, optimizing cylinder usage and functioning even at low pressures; (iv) as it is a parameter derived from pressure sensors, real-time monitoring platforms capable of seamless integration with embedded valve actuation and control systems can be easily employed.

## 6. Conclusions

This paper introduced a micro-controlled air–O2 blender set to transform oxygen delivery in mechanical ventilation, promising significant improvements in patient outcomes, cost reduction, and resource efficiency in healthcare systems worldwide. The blender’s key innovations include the use of three pressure sensors to determine oxygen saturation quickly and accurately, replacing costly flow sensors. Additionally, its integrated mathematical model optimizes gas cylinder utilization, potentially contributing to substantial cost savings across healthcare systems. Moreover, its effectiveness at low pressures addresses a critical limitation of existing models, benefiting patients globally who require specialized care.

During this investigation, numerous opportunities for further research on the subject have been uncovered. Firstly, the next step is to develop a tangible prototype, enabling practical experiments to validate and refine theoretical findings, providing comprehensive insights into the blender’s performance under various conditions. Secondly, the integration of artificial intelligence (AI) in the blender presents an exciting direction, potentially optimizing precision, automation, and adaptability to dynamic medical environments through machine learning algorithms. Lastly, incorporating an Internet of things (IoT)-based monitoring and tuning platform can enhance healthcare efficiency by allowing remote oversight, adjustments, and proactive maintenance. Together, these directions aim to advance the air–oxygen blender’s efficiency and adaptability and introduce intelligent healthcare solutions.

In conclusion, the impact of this subject of study extends beyond technical specifications, reaching toward a future where advanced technology enables us to provide fair and life-saving care to all, regardless of resource constraints.

## Data Availability

Data are contained within the article.

## Abbreviations

The following abbreviations are used in this manuscript:

ICU	Intensive Care Unit
PID	Proportional–integral–derivative
